# Mouse Model for ROS1-Rearranged Lung Cancer

**DOI:** 10.1371/journal.pone.0056010

**Published:** 2013-02-13

**Authors:** Yasuhito Arai, Yasushi Totoki, Hiroyuki Takahashi, Hiromi Nakamura, Natsuko Hama, Takashi Kohno, Koji Tsuta, Akihiko Yoshida, Hisao Asamura, Michihiro Mutoh, Fumie Hosoda, Hitoshi Tsuda, Tatsuhiro Shibata

**Affiliations:** 1 Division of Cancer Genomics, National Cancer Center Research Institute, Chuo-ku, Tokyo, Japan; 2 Division of Genome Biology, National Cancer Center Research Institute, Chuo-ku, Tokyo, Japan; 3 Division of Pathology and Clinical Laboratories, National Cancer Center Hospital, Chuo-ku, Tokyo, Japan; 4 Thoracic Surgery Division, National Cancer Center Hospital, Chuo-ku, Tokyo, Japan; 5 Division of Cancer Prevention Research, National Cancer Center Research Institute, Chuo-ku, Tokyo, Japan; Univesity of Texas Southwestern Medical Center at Dallas, United States of America

## Abstract

Genetic rearrangement of the *ROS1* receptor tyrosine kinase was recently identified as a distinct molecular signature for human non-small cell lung cancer (NSCLC). However, direct evidence of lung carcinogenesis induced by *ROS1* fusion genes remains to be verified. The present study shows that *EZR-ROS1* plays an essential role in the oncogenesis of NSCLC harboring the fusion gene. *EZR-ROS1* was identified in four female patients of lung adenocarcinoma. Three of them were never smokers. Interstitial deletion of 6q22–q25 resulted in gene fusion. Expression of the fusion kinase in NIH3T3 cells induced anchorage-independent growth *in vitro*, and subcutaneous tumors in nude mice. This transforming ability was attributable to its kinase activity. The ALK/MET/ROS1 kinase inhibitor, crizotinib, suppressed fusion-induced anchorage-independent growth of NIH3T3 cells. Most importantly, established transgenic mouse lines specifically expressing EZR-ROS1 in lung alveolar epithelial cells developed multiple adenocarcinoma nodules in both lungs at an early age. These data suggest that the *EZR-ROS1* is a pivotal oncogene in human NSCLC, and that this animal model could be valuable for exploring therapeutic agents against *ROS1*-rearranged lung cancer.

## Introduction

Lung cancer is the leading cause of cancer death around the world [1]. Lung adenocarcinoma (LADC), the most common form of non-small-cell lung cancer (NSCLC), comprises several different genomic subsets defined by unique oncogenic alterations, and a considerable proportion of LADC cases harbor driver alterations in the *EGFR, KRAS* and *ALK* genes at the mutually exclusive manner with rare exceptions [2]–[5]. Understanding the molecular basis of cancer allows us to develop therapeutic agents that target genetic druggable aberrations identified in cancer genomes. Tyrosine kinase inhibitors (TKIs) that target the EGFR and ALK proteins are particularly effective in the treatment of LADC carrying *EGFR* mutations and *ALK* fusions, respectively [2]–[6]. However, the development of an effective TKI requires experimental validation of the genetic aberrations as actionable and druggable. Transgenic mouse models harboring *EGFR* mutations or *EML4-ALK* gene fusions have successfully demonstrated the oncogenic potential of the alterations and the efficacy of TKI therapy [7], [8]. Genetic rearrangement of the *ROS1* was recently identified as a distinct molecular signature for human LADC [9]–[16]. In the present study, we established a mouse model of *ROS1* fusion, and showed that *EZR-ROS1* as an essential driver oncogene in lung carcinogenesis.

## Results

### Identification of EZR-ROS1 Fusion Gene in LADC of Never-smokers

**Figure 1:**
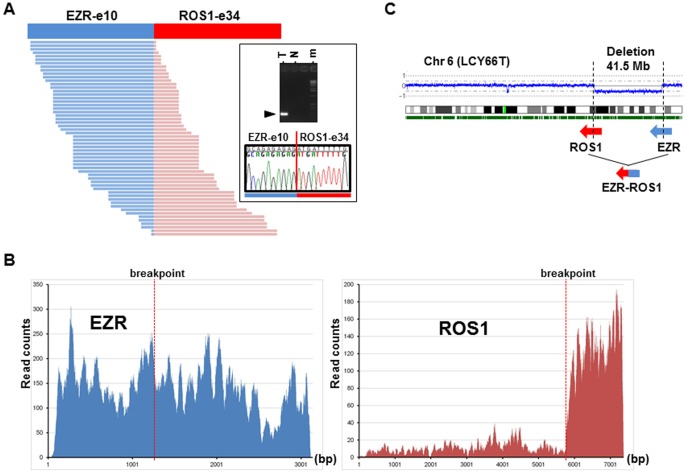
Identification of the *EZR-ROS1* fusion. (A) Junction reads representing *EZR-ROS1* fusion transcripts in LCY66T sample (left). Sanger sequencing of the RT-PCR product validated tumor-specific in-frame fusion transcript (right). m: molecular marker. (B) Expression profiles of *EZR* and *ROS1* in LCY66T. Active expression of the *ROS1* gene was observed after the fusion point. (C) SNP array CGH analysis of the LCY66T. Copy number throughout chromosome 6 is plotted as the log2 ratio.

Whole transcriptome high-throughput sequencing of tumor specimens is one of the most effective methods for identifying fusion oncogenes [17]. Analysis of five LADC cases of never-smokers without *EGFR/KRAS/ALK* alterations using transcriptome sequencing identified 56 reads overriding the in-frame *EZR-ROS1* gene fusion point connecting *EZR* exon 10 to *ROS1* exon 34 in one tumor. RT-PCR analysis of matched non-cancerous tissues confirmed tumor-specific expression of the fusion transcript (Figure 1A). In addition, transcriptome sequencing clearly demonstrated a specific increase in the expression of the fused 3′ portion of *ROS1* (exons 34 to 43) after the breakpoint, suggesting that the *EZR-ROS1* fusion transcript causes aberrant overexpression of *ROS1* tyrosine kinase domain along with the 5′ portion of *EZR* (Figure 1B). SNP array comparative genomic hybridization (array CGH) data showed that this fusion gene was generated by a large interstitial deletion spanning ∼41.5 Mb on chromosome 6q22-q25 (Figure 1C). Genomic PCR and sequencing analysis also revealed the deletion of 41.5 Mb causing somatic fusions of the *EZR* intron 10 at 6q25 with the *ROS1* intron 33 at 6q22 (Figure S1).

RT-PCR and Sanger sequencing analysis of 569 LADC specimens from Japanese individuals, including the above-mentioned cases (343 cases with early pathological stage and 226 cases with advanced stage), identified four cases harboring this fusion transcript (Figure S2). All four *EZR-ROS1* fusion-positive cases were female, and harbored neither *EGFR*/*KRAS/HER2* mutations nor *EML4-ALK/KIF5B-RET* fusions. Three cases were poorly differentiated adenocarcinomas of never smokers, and the other was a moderately differentiated adenocarcinoma of a smoker.

### Transforming Activity of EZR-ROS1

**Figure 2:**
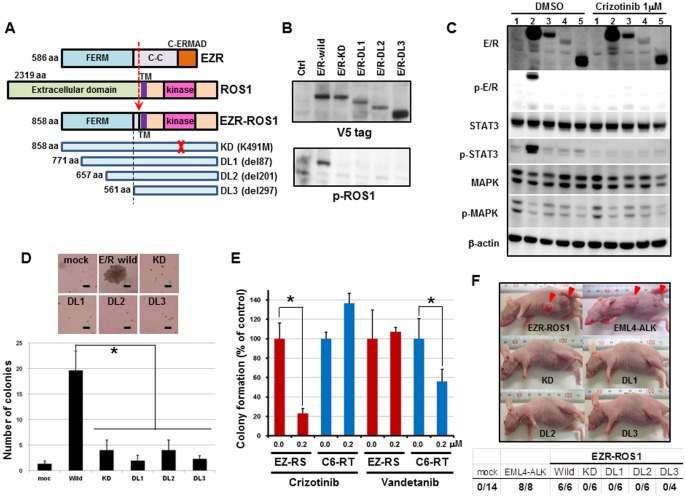
Oncogenic activity of the *EZR-ROS1* fusion gene. (A) Schematic representation of EZR, ROS1, EZR-ROS1, and deletions/mutations of EZR-ROS1 genes. The domain organization is shown. C-C: coiled-coil domain; TM: transmembrane; C-ERMAD: C-terminal ERM associated domain. (B) ROS1 phosphorylation in wild-type and mutant EZR-ROS1 (E/R)-expressing NIH3T3 clones. Cell lysates from each clone were immunoblotted with anti-V5-tag (top) and anti-phosphorylated ROS1 (Tyr-2274, bottom) antibodies. (C) Suppression of ROS 1 kinase activity of EZR-ROS1 by crizotinib inhibits STAT3 activation. NIH3T3 cells transfected with 1: empty vector, 2: wild-type EZR-ROS1, 3: KD 4: DL1, 5: DL3 were serum starved and treated for 2 hr with DMSO or 1 µM of crizotinib, and immunoblotted with the relevant antibodies. β-actin was used as a loading control. E/R: EZR-ROS1, p-E/R: phosphorylated EZR-ROS1 detected with an anti-phosphotyrosine-2274 antibody of ROS1. (D) Soft agar colony formation of wild-type and mutant EZR-ROS1 expressing NIH3T3 clones. A representative picture of colony formation for each clone is plotted at the top (scale bar, 100 µm). The number of colonies obtained for each clone is plotted at the bottom. *P<0.05. (E) Crizotinib-induced suppression of anchorage-independent growth of NIH3T3 cells expressing EZR-ROS1. Bar graph showing the percentage of NIH3T3 colonies induced by *EZR-ROS1* or *CCDC6-RET* after treatment with 200 nM of crizotinib or vandetanib with respect to those formed by DMSO-treated cells. EZ-ROS: EZR-ROS1, C6-RET: CCDC6-RET. *P<0.05. (F) Representative pictures of mice subcutaneously transplanted with NIH3T3 cells expressing wild-type, kinase domain-mutated, or amino-terminal-deleted EZR-ROS1. An EML4-ALK-expressing NIH3T3 clone was used as a positive control. The number of tumors per injection in each transfectant is shown below the photographs.

*EZR-ROS1* cDNA isolated from the tumor specimen encoded a protein of 858 amino acids (Figure 2A; GenBank/DDBJ accession number AB698667). The protein connects the FERM domain [18] of ezrin (EZR) with the transmembrane and kinase domains of ROS1, but lacks most of the coiled-coil domain of EZR.

To examine the oncogenic activity of the *EZR-ROS1* fusion *in vitro*, we established stable NIH3T3 clones expressing wild-type EZR-ROS1 and kinase-dead mutant EZR-ROS1 (KD), in which the ATP-binding lysine residue was mutated to methionine (K491M), as well as mutants with serially deleted amino-terminal FERM domains (DL1, DL2 and DL3; Figure 2A). Autophosphorylation of specific tyrosine residues is a crucial event in the activation of distinct signal transduction pathways, and Tyr-2274 of ROS1 is a specific autophosphorylation site essential to induce kinase activity for transformation [19]. In transformation assays, phosphorylation of the Tyr-2274 (corresponding to Tyr-785 in wild type EZR-ROS1 fusion) was observed in a wild-type EZR-ROS1-expressing clone, but was not detected in kinase-dead (KD) and deleted (DL) mutants; this implies that the amino-terminal portion of FERM (1–88 amino acids) is necessary for ROS1 kinase activation (Figure 2B). Wild-type *EZR-ROS1* but not KD/DL mutants specifically induced activation of STAT3 for downstream signaling, and produced significantly anchorage-independent growth (Figure 2C, D). The anchorage-independent growth induced by *EZR-ROS1* was suppressed by treatment with crizotinib, a TKI against ALK/MET/ROS1, whereas the growth induced by another oncogene of lung, *CCDC6-RET* [11] was not (Figure 2E). On the contrary, vandetanib, a TKI against RET/EGFR/VEGFR was effective in inhibiting the colony formation of CCDC6-RET expressing cells, but not in the EZR-ROS1 expressing cells. As shown in Figure 2C, crizotinib treatment suppressed phosphorylation of EZR-ROS1, and inhibit the activation of STAT3.

Next, the NIH3T3 cells were subcutaneously injected into immune-compromised mice. Wild-type EZR-ROS1-expressing clones invariably produced tumors (6/6), while none of the KD and DL mutants-expressing clones produced tumors (Figure 2F), confirming that *in vivo* tumorigenic activity of *EZR-ROS1* requires ROS1 kinase activity.

### Development of LADC in EZR-ROS1 Transgenic Mice

**Figure 3:**
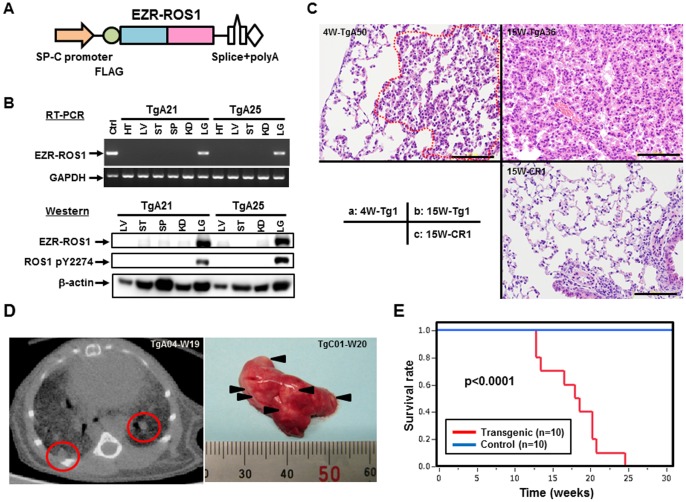
Alveolar epithelium-specific EZR-ROS1 expression generates lung adenocarcinoma *in vivo*. (A) Schematic presentation of the *SP-C/EZR-ROS1/polyA* transgene. (B) Expression of the exogenous *EZR-ROS1* gene in transgenic mice. RT-PCR (top) and immunoblot analysis (bottom) of mouse tissues revealed that EZR-ROS1 was specifically expressed in the lungs of two transgenic mice (TgA21 and TgA25). HT: heart, LV: liver, ST: stomach, SP: spleen, KD: kidney, LG: lung (C) Representative histological analysis of lung lesions in transgenic mice. Hematoxylin-eosin staining shows wide-spread lesions in both 4-week-old and 15-week-old fusion-positive mice. Tg: fusion-positive, CR: fusion-negative. Scale bar, 100 µm. (D) Computed tomography (left) of lungs in TgA04 mouse at week 19. Enhanced lesions in both lungs were detected. Multiple nodular lesions (right) were observed on the pleural surface of the lung in TgC01 mouse at necropsy. (E) Survival curves for transgenic and control mice generated using the Kaplan-Meier method.

To further evaluate the role of *EZR-ROS1* in lung carcinogenesis, we generated transgenic mice expressing the fusion gene under the control of a type 2 alveolar epithelium-specific surfactant C gene promoter [20] (Figure 3A). We obtained four independent lines (TgA, B, C and D) with different copy number of the transgene (Figure S3) and detected lung adenocarcinoma nodules in all lines examined except TgD. Analysis of fusion protein expression level among them revealed no expression in TgD (Figure S4). The birth rate of transgene-positive progenies was low in TgC (Transgene-positive F1 progeny number : total F1 number; 1∶3), and we failed to keep up a TgC line, then we mainly analyzed one line (TgA), which harbors approximately four copies of the transgene. RT-PCR and immunoblot analysis verified lung-specific *EZR-ROS1* mRNA and protein expression, and indicated phosphorylation of the EZR-ROS1 fusion protein (Figure 3B). Although endogenous *Ezrin* was ubiquitously expressed in many tissues, endogenous *Ros1*-transcript was detected only in stomach, kidney and lung. Protein expression levels of endogenous ROS1 were very weak compared with the levels of the fusion gene in the transgenic mice (Figure S4). Even at the four-week-old, multiple lesions over 1 mm in diameter were detected in the transgenic mice, and tumors occupied over 40% of sectioned surface of lung (Figure 3C and Figure S5). Computed tomography examination detected multiple nodules in both lungs, and the mice showed reduced survival (Figure 3D, E). Histological examination of lung tumors in the transgenic mouse lines generally demonstrated adenocarcinomas with papillary/lepidic growth pattern (Figure 3C). These lesions were shown to be invasive adenocarcinomas with moderate mitotic activity as revealed by positive Ki-67 staining (Figure S6A). However, in some cases of TgB lines, we observed accumulation of cytoplasmic mucin in tumor cells (Figure S6B).

Despite the presence of multiple tumors in the lungs of the transgenic mice, we failed to detect distant metastasis at necropsy in TgA, B and C mice. Thus, it is likely that expression of *EZR-ROS1* alone is not sufficient to render the cancer cells metastatic.

## Discussion

The present study identified *EZR-ROS1* as a pivotal driver oncogene in lung carcinogenesis. Ezrin is ubiquitously expressed in many tissues. In the *EZR-ROS1* fusion detected by RNA sequencing of LADC cases, 5′ portion of *EZR* causes aberrant overexpression of kinase domain of *ROS1*. No evident effect to the transcript levels of the 3′ portion of *EZR* was observed. This might be ascribable to the excess expression of the wild type *EZR* over the fusion gene. We also revealed that ROS1 kinase activation in this fusion requires the N-terminal FERM domain of EZR. FERM associates with many different proteins including phospholipids, the scaffolding proteins EBP50 and E3KARP, and other membrane-associated proteins that may regulate the dimerization or oligomerization of ezrin [21]. Many fusion kinase proteins, including ALK and RET, display constitutive tyrosine kinase activity attributable to dimerization domains in the amino-terminal fusion partner [6], [22]. However, another ROS1 fusion protein, FIG-ROS1, which is found in human glioblastoma, cholangiocarcinoma and lung adenocarcinoma, showed no dimerization properties, instead existing as a monomer in the fusion protein despite retaining the coiled-coil domains and a leucine zipper [19]. Therefore, the molecular mechanisms underlying ROS1 activation by the FERM domain remains unclear.

The transgenic mice showed an emergence of multiple adenocarcinoma nodules at an early point, and the fast progression of the tumors. These features are broadly similar to the *EML4-ALK* mouse model [8]. Several groups reported that mucinous cribriform pattern and signet ring cell are characteristic histological features of E*ML4-ALK* positive human lung cancer [23]–[25]. Recently, we investigated histopathology of *ROS1*-fusion positive human lung cancers [16]. Although other researchers reported that signet ring cell feature was not common in *ROS1*-rearranged lung cancers [10], we found that 53% of the cases harbored mucinous cribriform or signet ring cell features similar to the *ALK*-rearranged lung cancers but that the rest showed papillary/lepidic growth pattern. *EZR-ROS1*-positive tumors seemed less well differentiated, and showed more frequently histological features of mucinous cribriform or signet ring cell. Our mouse model of *EZR-ROS1* lung cancer generally demonstrated papillary/lepidic growth pattern, but in some cases, we observed accumulation of cytoplasmic mucin in tumor cells, which quite resembles to the characteristic histology reported in *ROS1*-rearranged lung cancer. Currently we have no answer why only part of mice harbored tumors with mucin accumulation.

The *EZR-ROS1* fusion gene was specifically detected in lung cancer specimens of female never-smokers without *EGFR, KRAS,* and *ALK* alterations. It was estimated that ∼2% of patients in White and Asian lung cancer cohorts had *ROS1*-rearrangements, which occur at significantly higher rates in younger, non-smoking, female individuals [10], [11], [16]. Although each alteration is infrequent, *ROS1* fusions with many kinds of 5′ partner genes (*CCDC6, CD74, EZR, FIG, KDELR2, LRIG3, SDC4, SLC34A2 and TPM3*) have been reported in lung, brain, biliary tract, and ovarian cancers [9]–[16], [26]–[28]. These *ROS1*-rearranged tumors could be targeted therapeutically with specific kinase inhibitors, including crizotinib [10], [14], [27], [29]. Two LADC patients had a remarkable clinical response to crizotinib [10], [14]. Thus, our *EZR-ROS1* lung cancer animal model could be valuable for evaluating the therapeutic potential of these compounds and novel drugs as well as biological features of *ROS1*-rearranged lung cancer *in vivo*.

## Materials and Methods

### Clinical Samples

Tissue specimens from lung cancer patients were provided by the National Cancer Center Biobank, Japan. High-molecular weight genomic DNA and RNA were extracted from fresh frozen tumor specimens and non-cancerous lung tissues. Written informed consent was obtained from each patient. The study protocol was approved by the Ethical Committee of National Cancer Center, Tokyo, Japan.

### Analysis of Whole-transcriptome Sequence Data

Insert cDNA libraries (150–200 bp) were prepared from 2 µg of total RNA using the mRNAseq Sample Preparation Kit (Illumina). The libraries were subjected to paired-end sequencing of 50 bp on the HiSeq2000 (Illumina), according to the manufacturer’s instructions. Paired-end reads were mapped to known RNA sequences in the RefSeq, Ensembl, and LincRNA databases using the Bowtie program as described previously [30].

### RT-PCR, Genomic PCR and Sequencing

Total RNA was reverse-transcribed to cDNA using Superscript III (Life Technologies). cDNA or genomic DNA was subjected to PCR amplification using Ex-Taq (Takara Bio) and primers EZR-e10-CF1 (GAAAAGGAGAGAAACCGTGGAG) and ROS1-e34-CR1 (TCAGTGGGATTGTAACAACCAG). The PCR products were directly sequenced by Sanger sequencing using the BigDye terminator kit (Life Technologies).

### SNP Array CGH Analysis

Chromosomal copy number for the tumors was determined using high-resolution SNP arrays (GeneChip Mapping 250K-Nsp array, Affymetrix). Genomic DNA was labeled and hybridized to the SNP arrays according to the manufacturer’s instructions, and copy numbers were calculated from the hybridization signals using the CNAG program [31].

### Vector Cloning, and Generation of Deletion and Point Mutants

The coding region of *EZR-ROS1* cDNA was obtained by PCR amplification from LCY66 tumor cDNA using Phusion Taq polymerase (New England Biolabs) and primers EZR-H1F1 (CACCATGCCGAAACCAATCAATGTCCGAGTT) and ROS1-H1R1 (ATCAGACCCATCTCCATATCCACTGTG). *EML4-ALK* cDNA and *CCDC6-RET* cDNA were amplified from an *EML4-ALK*-positive primary lung cancer sample (E13;A20) and from a *CCDC6-RET*-positive primary lung cancer sample (C1;R12), respectively. The PCR products were subcloned into a pcDNA3.1D-V5-His plasmid (Life Technologies). Replacement of lysine with methionine at codon 491 in the *EZR-ROS1* gene was performed using a PrimeSTAR site-directed mutagenesis kit (Takara Bio). N-terminal deletion mutants of the FERM domain of *EZR-ROS1* cDNA were constructed by PCR using the primers EZR-FERM-AF (CACCATGGTGGCTGAGGAGCTCATCCAGGACATC) and ROS1-H1R1 for DL1, EZR-FERM-BF (CACCATGATCAACTATTTCGAGATAAAAAACAAG) and ROS1-H1R1 for DL2, and EZR-FERM-CF (CACCATGACCATCGAGGTGCAGCAGATGAAGGC) and ROS1-H1R1 for DL3. The plasmids were transfected into NIH3T3 cells using Lipofectamine 2000 reagent (Life Technologies), and stable clones were isolated by G418 selection (0.7 mg/ml). For the colony formation assay, cells were embedded and cultured in 0.4% soft agar in triplicate and the number of colonies was counted after 21 days. Quantification of anchorage–independent growth under the condition with or without crizotinib (S1068, Selleck) and vandetanib (S1046, Selleck) after 9 days was performed with CytoSelect-96 kit (Cell Biolabs). The compound solution was added to the top layer of soft agar every 3 days.

### Immunoblot Analysis

Whole cell lysates were extracted with CelLytic M reagent (#C2978, Sigma), and subjected to SDS-PAGE followed by blotting onto a PVDF membrane. Detection of Western blots was performed with the WesternBreeze Chemiluminescent Immunodetection kit (Life Technologies) using primary antibodies against ROS1 (#9202, Cell Signaling Technology), phosphorylated-ROS1 (Tyr2274) (#3078, Cell Signaling Technology), STAT3 (#610189, BD), phosphorylated-STAT3 (Tyr705) (#9138, Cell Signaling Technology), p44/42 MAPK (#4695, Cell Signaling Technology), phosphorylated-p44/42 MAPK (Thr202/Tyr204) (#9106, Cell Signaling Technology), Ezrin (#4135, Cell Signaling Technology), p53 (#6243, Santa Cruz), and b-actin (#A5441, Sigma).

### Suppression of ROS 1 Kinase Activity of EZR-ROS1 by Crizotinib

Transfected NIH3T3 cells (empty vector, wild-type EZR-ROS1, KD/DL mutants) were serum starved for 2 hr, then added for 2 h with 1% DMSO or 1 µM crizotinib, then the culture medium were changed with standard 10% FBS medium for 10 min. Whole cell lysates were subjected to immunoblot analysis.

### Subcutaneous Transplantation in Immune-compromised Mice

A total of 1×10^6^ cells were injected subcutaneously into nude mice (BALB/c-nu/nu, CLEA Japan). Mice were monitored daily for tumor formation. All animal procedures were performed with the approval of the animal ethical committee of the National Cancer Center.

### Generation and Examination of *EZR-ROS1* Transgenic Mice

FLAG-tagged *EZR-ROS1* cDNA was subcloned into an *SPC-iNOS* plasmid (provided by Dr. Hagiwara), which included an *SPC* promoter and a polyadenylation signal, by replacing the *iNOS* fragment with the cDNA. The expression cassette with the *SPC* promoter was excised from the construct and injected into pronuclear-stage embryos of C57BL/6J mice (Unitech Japan). The copy number of the transgene was determined by Southern blot analysis of DNA from the tails of animals. Transgenic lines were maintained by backcrossing to C57BL/6 mice. Total RNA was isolated from the organs of transgenic mice and subjected to RT-PCR analysis to detect *EZR-ROS1*, endogenous *Ros1*, endogenous *Ezrin* and *Gapdh* mRNAs. To detect EZR-ROS1 protein, endogenous ROS1 and Ezrin in tissues, lysed homogenates were subjected to immunoblot analysis using anti-ROS1, anti-Ezrin and anti-β-actin antibodies. Examination of lung tumors in live animals was performed with an X-ray CT apparatus (eXplore micro-CT, GE Healthcare). Lung tissues were fixed in 10% formalin and paraffin-embedded. Hematoxylin-Eosin staining and immunohistochemistry for Ki67 was performed as previously described [32].

## Supporting Information

Figure S1 **Detection of** ***EZR-ROS1*** **genomic breakpoint junction.** Electropherogram for Sanger sequencing of genomic fragments encompassing the *EZR-ROS1* breakpoint junction of LCY66 tumor. Genomic PCR products amplified by the EZR-e10-CF1 and ROS1-e34-CR1 primers were directly sequenced using the EZR-e10-CF1 primer. Numbers above the electropherogram indicate genomic position in chromosome 6 (human genome build 37.3). A genomic fragment of 35 bp of *EZR* intron 10 was inverted within the intron before the fusion to *ROS1* intron 33.
(PDF)

Figure S2 **Detection of fusion gene transcripts in clinical samples by RT-PCR.** Representative RT-PCR results showing fusion-positive and fusion-negative cases using primers EZR-e10-CF1 and ROS1-e34-CR1. M:molecular marker, NC: negative control. RT-PCR for wild-type *EZR* transcript (primers EZR-e4-CF1 and EZR-e7-CR1) and for *GAPDH* (primers for GAPDH-F and GAPDH-R) is also shown.
(PDF)

Figure S3 **Copy number analysis of the transgene in transgenic mice.** Genomic DNA was isolated from the tails of transgenic mice generated from pronuclear-stage C57BL/6J embryos. This gDNA was then subjected to Southern blot analysis with a PCR-amplified SPC promoter fragment of 464 bp, generated using primers SPC-pro-F and SPC-pro-R, as a probe. Control samples on the right were comprised of mouse genomic DNA with the indicated copies of the transgene per diploid genome. The ID numbers of mice positive for the transgene are shown at the top.
(PDF)

Figure S4 **Gene expressions in transgenic mice.** Expression of the genes indicated at left side was investigated by RT-PCR or immunoblot analysis. In RT-PCR, PCR cycles to amplify target genes were indicated at right side. Ezrin showed ubiquitous endogenous expression, however endogenous Ros1 expression was low. No expression of EZR-ROS1 fusion protein was detected in TgD line mice (*). SW480 was used as a negative control for fusion expression. HT: heart, LV: liver, ST: stomach, SP: spleen, KD: kidney, LG: lung.
(PDF)

Figure S5 **Lung tumor development in transgenic mice.** Lung tissues of TgA mice were cross-sectioned and histologically characterized. The number and size of lesions were surveyed in fusion-positive mice (Tg) and fusion-negative mice (CR) at 4 weeks and 15 weeks after birth. (a) Tumor lesions were classified along its size in diameter (mm), and counted. (b) Tumor occupancy was calculated from the deduced tumor area.
(PDF)

Figure S6 **Histological characterization of lung tumors in transgenic mice.** (A) Hematoxylin-eosin staining of a mouse lung showing invasive lung adenocarcinoma surrounding a pulmonary vessel (a1). Higher magnification of the tumor (a2). Positive Ki-67 staining in the tumor (a3). Scale bar, 100 µm. (B) Hematoxylin-eosin staining of a mouse lung showing cytoplasmic mucin in lung adenocarcinoma cells (b1). Higher magnification of the tumor (b2). Scale bar, 200 µm.
(PDF)
