# Supplementary material for: Mouse Model for ROS1-Rearranged Lung Cancer
Source: PLoS One. 2013 Feb 13;8(2):e56010. doi: 10.1371/journal.pone.0056010 (PMC3572153; doi:10.1371/journal.pone.0056010)
Supplement: Figure S1 — Detection of EZR-ROS1 genomic breakpoint junction. Electropherogram for Sanger sequencing of genomic fragments encompassing the EZR-ROS1 breakpoint junction of LCY66 tumor. Genomic PCR products amplified by the EZR-e10-CF1 and ROS1-e34-CR1 primers were directly sequenced using the EZR-e10-CF1 primer. Numbers above the electropherogram indicate genomic position in chromosome 6 (human genome build 37.3). A genomic fragment of 35 bp of EZR intron 10 was inverted within the intron before the fusion to ROS1 intron 33. (PDF) [file pone.0056010.s001.pdf]

Supplemental Figure

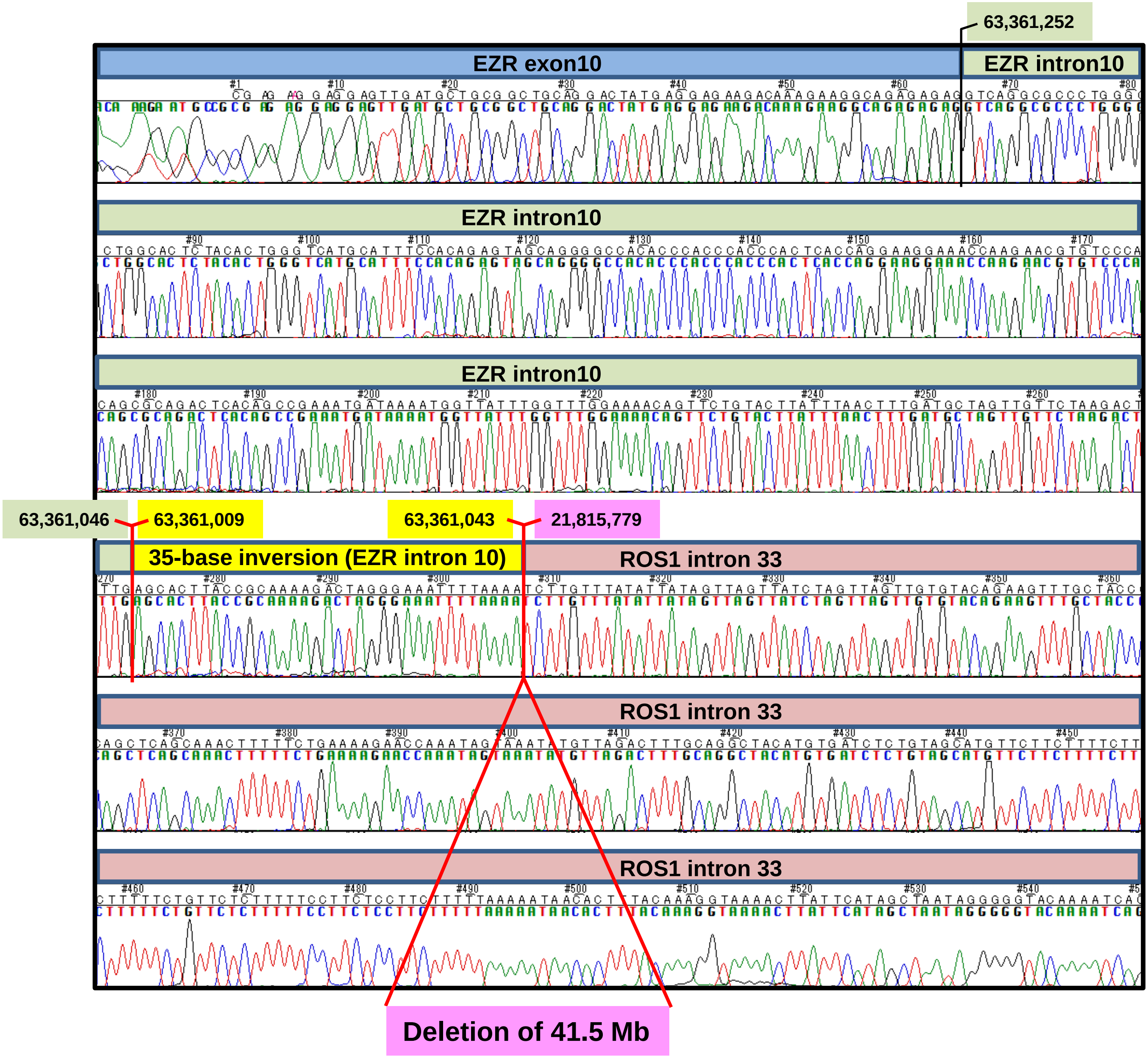

Figure S1 Detection of EZR-ROS1 genomic breakpoint junction.
Electropherogram for Sanger sequencing of genomic fragments encompassing the EZR-ROS1 breakpoint junction of LCY66 tumor. Genomic PCR products amplified by the EZR-e10-CF1 and ROS1-e34-CR1 primers were directly sequenced using the EZR-e10-CF1 primer. Numbers above the electropherogram indicate genomic position in chromosome 6 (human genome build 37.3). A genomic fragment of 35 bp of EZR intron 10 was inverted within the intron before the fusion to ROS1 intron 33.

Primers:
EZR-e10-CF1: GAAAAGGAGAGAAACCGTGGAG
ROS1-e34-CR1: TCAGTGGGATTGTAACAACCAG
