# Supplementary material for: Mouse Model for ROS1-Rearranged Lung Cancer
Source: PLoS One. 2013 Feb 13;8(2):e56010. doi: 10.1371/journal.pone.0056010 (PMC3572153; doi:10.1371/journal.pone.0056010)
Supplement: Figure S2 — Detection of fusion gene transcripts in clinical samples by RT-PCR. Representative RT-PCR results showing fusion-positive and fusion-negative cases using primers EZR-e10-CF1 and ROS1-e34-CR1. M:molecular marker, NC: negative control. RT-PCR for wild-type EZR transcript (primers EZR-e4-CF1 and EZR-e7-CR1) and for GAPDH (primers for GAPDH-F and GAPDH-R) is also shown. (PDF) [file pone.0056010.s002.pdf]

Supplemental Figure

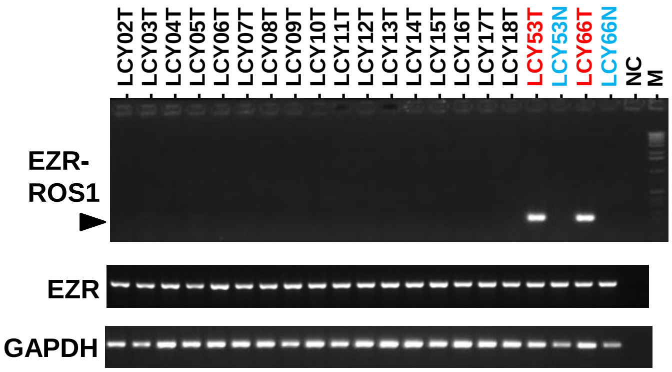

Figure S2 Detection of fusion gene transcripts in clinical samples by RT-PCR. Representative RT-PCR results showing fusion-positive and fusion-negative cases using primers EZR-e10-CF1 and ROS1-e34-CR1. M:molecular marker, NC: negative control. RT-PCR for wild-type EZR transcript (primers EZR-e4-CF1 and EZR-e7-CR1) and for GAPDH (primers for GAPDH-F and GAPDH-R) is also shown.

Primers:
EZR-e04-CF1: GTACTTTGGCCTCCACTATGT
EZR-e07-CR1: ACTCCAAGCCAAAGGTCTGTT
EZR-e10-CF1: GAAAAGGAGAGAAACCGTGGAG
ROS1-e34-CR1: TCAGTGGGATTGTAACAACCAG
GAPDH-F: TGGAAATCCCATCACCATCT
GAPDH-R: GTCTTCTGGGTGGCAGTGAT
