# Supplementary material for: Mouse Model for ROS1-Rearranged Lung Cancer
Source: PLoS One. 2013 Feb 13;8(2):e56010. doi: 10.1371/journal.pone.0056010 (PMC3572153; doi:10.1371/journal.pone.0056010)
Supplement: Figure S3 — Copy number analysis of the transgene in transgenic mice. Genomic DNA was isolated from the tails of transgenic mice generated from pronuclear-stage C57BL/6J embryos. This gDNA was then subjected to Southern blot analysis with a PCR-amplified SPC promoter fragment of 464 bp, generated using primers SPC-pro-F and SPC-pro-R, as a probe. Control samples on the right were comprised of mouse genomic DNA with the indicated copies of the transgene per diploid genome. The ID numbers of mice positive for the transgene are shown at the top. (PDF) [file pone.0056010.s003.pdf]

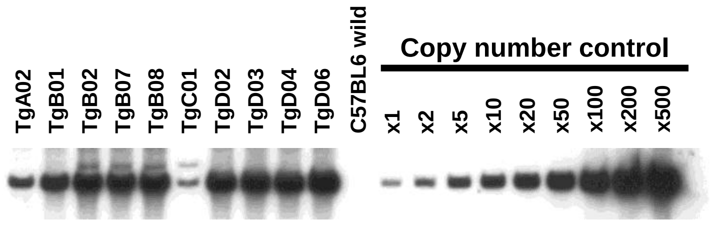

Figure S3 Copy number analysis of the transgene in transgenic mice.
Genomic DNA was isolated from the tails of transgenic mice generated from pronuclear-stage C57BL/6J embryos. This gDNA was then subjected to Southern blot analysis with a PCR-amplified SPC promoter fragment of 464 bp, generated using primers SPC-pro-F and SPC-pro-R, as a probe. Control samples on the right were comprised of mouse genomic DNA with the indicated copies of the transgene per diploid genome. The ID numbers of mice positive for the transgene are shown at the top.

Primers:
SPC-pro-F: TGACCTCGTAAATACATAGAGATGG
SPC-pro-R: TCAAGTACTCTTTCCCTTACCTTGC
