# Supplementary material for: Mouse Model for ROS1-Rearranged Lung Cancer
Source: PLoS One. 2013 Feb 13;8(2):e56010. doi: 10.1371/journal.pone.0056010 (PMC3572153; doi:10.1371/journal.pone.0056010)
Supplement: Figure S4 — Gene expressions in transgenic mice. Expression of the genes indicated at left side was investigated by RT-PCR or immunoblot analysis. In RT-PCR, PCR cycles to amplify target genes were indicated at right side. Ezrin showed ubiquitous endogenous expression, however endogenous Ros1 expression was low. No expression of EZR-ROS1 fusion protein was detected in TgD line mice (*). SW480 was used as a negative control for fusion expression. HT: heart, LV: liver, ST: stomach, SP: spleen, KD: kidney, LG: lung. (PDF) [file pone.0056010.s004.pdf]

Supplemental Figure

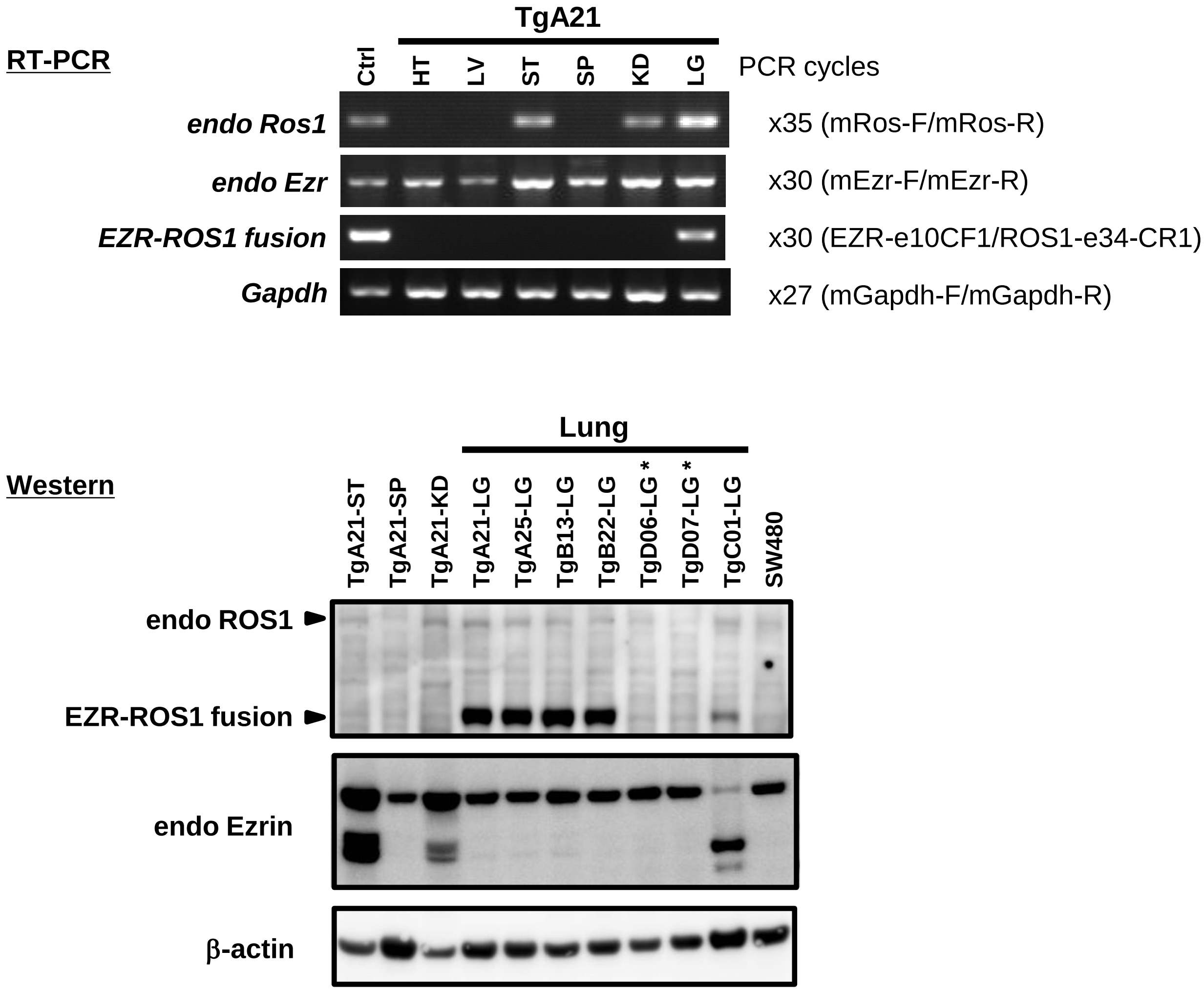

Figure S4 Gene expressions in transgenic mice.
Expression of the genes indicated at left side was investigated by RT-PCR or immunoblot analysis. In RT-PCR, PCR cycles to amplify target genes were indicated at right side. Ezrin showed ubiquitous endogenous expression, however endogenous Ros1 expression was low. No expression of EZR-ROS1 fusion protein was detected in TgD line mice (*). SW480 was used as a negative control for fusion expression. HT: heart, LV: liver, ST: stomach, SP: spleen, KD: kidney, LG: lung.

RT-PCR Primers:
mRos-F: TTTCGAGTGGTTTGGATCTTC
mRos-R: GATAGAAAACCTGTAGGTGG
mEzr-F: ACGAGGAGAAGCGGATCACA
mEzr-R: AATCTGACCTGTTTGGCACTA
EZR-e10-CF1: GAAAAGGAGAGAAACCGTGGAG
ROS1-e34-CR1: TCAGTGGGATTGTAACAACCAG
mGapdh-F: AGCTTGTCATCAACGGGAAG
mGapdh-R: CCTGCTTCACCACCTTCTTG
