# Supplementary material for: Mouse Model for ROS1-Rearranged Lung Cancer
Source: PLoS One. 2013 Feb 13;8(2):e56010. doi: 10.1371/journal.pone.0056010 (PMC3572153; doi:10.1371/journal.pone.0056010)
Supplement: Figure S5 — Lung tumor development in transgenic mice. Lung tissues of TgA mice were cross-sectioned and histologically characterized. The number and size of lesions were surveyed in fusion-positive mice (Tg) and fusion-negative mice (CR) at 4 weeks and 15 weeks after birth. (a) Tumor lesions were classified along its size in diameter (mm), and counted. (b) Tumor occupancy was calculated from the deduced tumor area. (PDF) [file pone.0056010.s005.pdf]

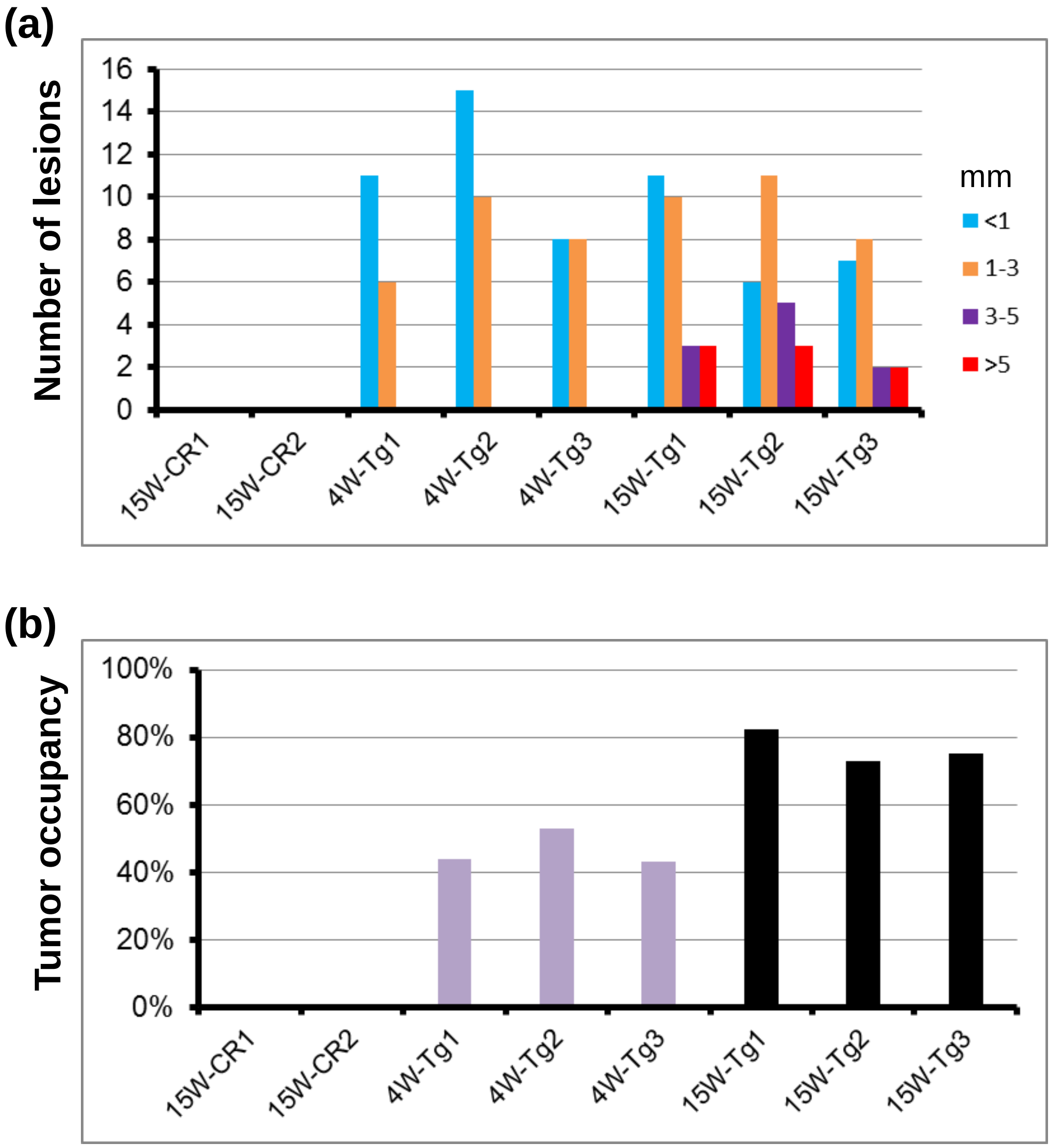

Figure S5 Lung tumor development in transgenic mice.
Lung tissues of TgA mice were cross-sectioned and histologically characterized. The number and size of lesions were surveyed in fusion-positive mice (Tg) and fusion-negative mice (CR) at 4 weeks and 15 weeks after birth. (a) Tumor lesions were classified along its size in diameter (mm), and counted. (b) Tumor occupancy was calculated from the deduced tumor area.
