# Supplementary material for: Mouse Model for ROS1-Rearranged Lung Cancer
Source: PLoS One. 2013 Feb 13;8(2):e56010. doi: 10.1371/journal.pone.0056010 (PMC3572153; doi:10.1371/journal.pone.0056010)
Supplement: Figure S6 — Histological characterization of lung tumors in transgenic mice. (A) Hematoxylin-eosin staining of a mouse lung showing invasive lung adenocarcinoma surrounding a pulmonary vessel (a1). Higher magnification of the tumor (a2). Positive Ki-67 staining in the tumor (a3). Scale bar, 100 µm. (B) Hematoxylin-eosin staining of a mouse lung showing cytoplasmic mucin in lung adenocarcinoma cells (b1). Higher magnification of the tumor (b2). Scale bar, 200 µm. (PDF) [file pone.0056010.s006.pdf]

Supplemental Figure

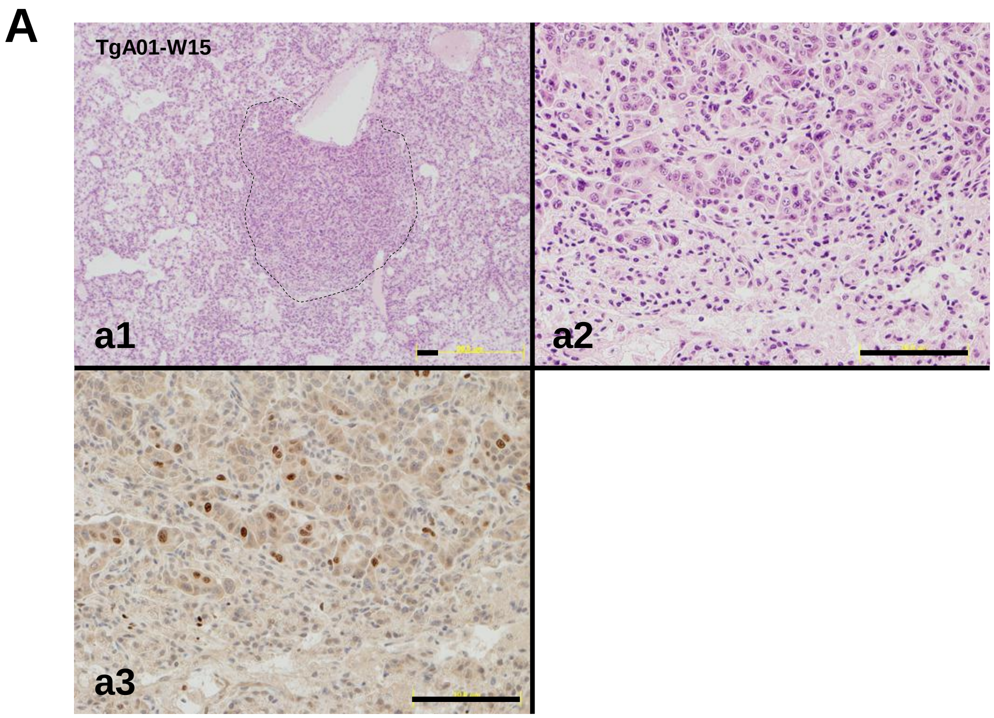

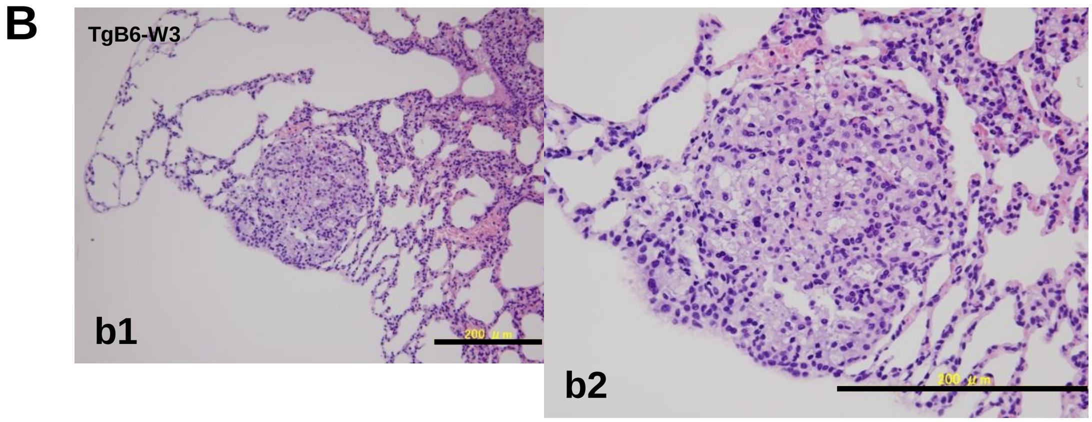

Figure S6 Histological characterization of lung tumors in transgenic mice.

(A) Hematoxylin-eosin staining of a mouse lung showing invasive lung adenocarcinoma surrounding a pulmonary vessel (a1). Higher magnification of the tumor (a2). Positive Ki-67 staining in the tumor (a3). Scale bar, 100μm.

(B) Hematoxylin-eosin staining of a mouse lung showing cytoplasmic mucin in lung adenocarcinoma cells (b1). Higher magnification of the tumor (b2). Scale bar, 200μm.
